# Associations among green spaces, ambient temperature, air pollution, and body mass index: a nationwide study in South Korea from 2008 to 2021

**DOI:** 10.1186/s12889-025-23390-z

**Published:** 2025-07-02

**Authors:** Yujin Song, Hoyoung Cha, Jongjin Baik, Changhyun Jun, Seokjoong Kim, Minjeong Kim, Sun-Young Jung

**Affiliations:** 1 Department of Family Medicine, National Medical Center, Seoul, Republic of Korea; 2 Department of Civil, Environmental and Architectural Engineering, Korea University, Seoul, Republic of Korea; 3 Future and Fusion Lab of Architectural, Civil and Environmental Engineering, Korea University, Seoul, Republic of Korea; 4 School of Civil, Environmental and Architectural Engineering, Korea University, Seoul, Republic of Korea; 5 College of Pharmacy, Chung-Ang University, Seoul, Republic of Korea

**Keywords:** Green space, Ambient temperature, Air pollution, Body mass index, Obesity

## Abstract

**Background:**

In this study, we explored the associations among green space, ambient temperature, and air pollution from 2008 to 2021, with the aim to provide insights into the trends and relationships among built and natural environments factors and their impact on obesity in South Korean adults.

**Methods:**

A total of 674,962 subjects from the Community Health Survey were analyzed. Descriptive statistics, Pearson correlation, and robust regression were used to help understand the changes in trend and the correlations between variables.

**Results:**

The mean age of the study subjects did not differ significantly based on body mass index (BMI); however, the obesity class 3 group had the lowest mean age. Exploration of green space and BMI showed a generally negative correlation in the normal weight group, but no such correlation was found in the overweight or obesity class 3 group. A positive correlation was observed between ambient temperature and BMI in the normal weight, overweight, and obesity class 1 groups. No strong association was found between air pollution and BM. While supporting evidence was found for the relationship between green space and ambient temperature, no such relationship was found between green space and air pollution. Lastly, air pollution and ambient temperature showed negative correlations, which contradicts the findings of prior studies.

**Conclusion:**

Further exploration is needed to identify potential mechanisms and develop policies and neighborhood-level interventions aimed at addressing the built and natural environments factors contributing to obesity in urbanized settings.

**Trial registration:**

The study design and data analysis protocol were reviewed and approved by the Institutional Review Board of the National Medical Center (IRB No. 202306069 IRB examination exemption approval 20230622).

## Background

Obesity is a significant public health threat that poses an immense challenge for physicians, scientists, and policymakers worldwide. According to the World Obesity Atlas 2024, an estimated 2.2 billion adults are currently overweight (BMI ≥ 25 to 30 kg/m²) or obese (BMI ≥ 30 kg/m²), and this number is projected to rise to nearly 3.3 billion by 2035, reflecting an increase from 42% to over 54% of adults [1]. In 2015, high body mass index (BMI) was reported to be responsible for 4 million deaths worldwide, which represented 7.1% of the deaths from any cause. Globally, 41% of BMI-related deaths and 34% of BMI-related disability-adjusted life-years were due to cardiovascular disease. Furthermore, high BMI also accounted for 28.6 million years lived with disability, which accounted for 3.6% of years lived with disability due to any cause globally [2]. Although Korea is not among the top countries with the fastest-growing proportion of adults with high BMI, the obesity rate among adults was 37.2% in 2022. The projected annual growth rate of adults with high BMI between 2020 and 2035 is 1.9%, signaling a concerning trend for the future [3]. Considering the enormous prevalence of overweight and obesity worldwide and its impact on both individuals and society, it is essential to identify its causes and develop sustainable prevention and reduction strategies.

To effectively address this global challenge, it is crucial to understand the multifaceted nature of obesity. Obesity is a chronic multifactorial condition that is influenced by a range of causes and determinants, including genetics, biological mechanisms, mental health, socioeconomic factors, commercial factors, healthcare policies, and environmental factors [4]– [5]. Recent epidemiological research has placed obesity within a larger socioecological context, emphasizing the role of built and natural environmental factors in shaping behaviors susceptible to obesity [6]. The built environment, which includes all human-made or modified aspects of an individual’s surroundings, including buildings, parks, facilities, and infrastructure, has become a focal point in obesity research, as most individuals spend the majority of their time in such settings. Understanding how these built environment influence physical activity and other health-related behaviors is essential for developing strategies to prevent and reduce obesity. 

According to Frank et al., there are two primary pathways through which the built and natural environments can influence health outcomes: one through behavior and the other through direct exposure [7]. The former pathway encompasses earlier studies that have explored how urban natural environments can promote physical activity, while the latter involves biological responses to environmental exposures, such as changes in ambient temperature that may affect individuals’ metabolism and energy use [7]. Moreover, these pathways are not mutually exclusive, which adds complexity to research on built and natural environments.

Despite this complexity, interest in the impact of the built and natural environments on health is continuously growing. Previous studies have predominantly focused on the built environment or the natural environment’s effects on our health [8–10]. Guo et al. (2020) statistically analyzed the association between long-term exposure to PM2.5 and obesity in children aged 6 to 17 over a five-year period in China. They found that PM2.5 exposure had a stronger association with childhood obesity in urban areas [8]. Moellering and Smith (2012) conducted a study examining the relationship between temperature and obesity. They noted that lower ambient temperatures increase energy expenditure to maintain body heat, whereas higher temperatures may suppress appetite. Thus, ambient temperature influences both energy intake and expenditure, which in turn can affect the occurrence of obesity [9]. Jia et al. (2021) systematically reviewed existing studies that analyzed the impact of green space accessibility on childhood obesity. Their study found a significant negative correlation between the risk of obesity and access to green space [10]. However, a limitation of these prior studies is that they tend to focus on a single environmental factor within urban areas and analyze relatively short time periods, despite the need to observe continuous urban influences. Furthermore, these previous studies had relaively modest sample sizes of approximately 40,000 participants, which may limit the generalizability of their findings.

Our study differs by aiming to integrate both built and natural environments and examine how their interplay may be associated with obesity. Given the complexity of these environment factors, this study has limited its scope to green space, ambient temperature, and air pollution, focusing on their impact on obesity among South Korean adults over an extended period from 2008 to 2021. These factors were selected due to their potential influence on physical activity, which in turn affects obesity. To address the complexity of environmental impacts, the scope was narrowed and specific aspects of each factor were considered. This approach allows to comprehensively examine the interplay between environment and their influence on obesity while maintaining a manageable scope for analysis. By doing so, our results aim toprovide insights into the current state of built and natural environmental factors and their impact on obesity in highly urbanized cities in South Korea, which will be of interest to clinicians, policymakers, urban planners, public health workers, and other medical professionals.

## Methods

### Study population

The baseline data of the study population were acquired from the Korea Community Health Survey (KCHS). The KCHS is conducted annually by the Korea Disease Control and Prevention Agency (KDCA) to collect information on households, socioeconomic status, health behaviors, medical use, and quality of life using computer-assisted face-to-face personal interviewing. The purpose of the KCHS is to provide autonomous local entity data that can be used to plan, implement, monitor, and evaluate community health [11]. The KCHS data can be requested through the website managed by the KDCA (https://chs.kdca.go.kr/) [12]. Access to the data is granted after the KDCA reviews the submitted data usage plan and approves the request. The raw data used in this study were from 2008 to 2021, covering all years from the initiation of the KCHS to the most recent data distributed.

The data collection for KCHS involved health centers located within cities, which played a vital role in generating the data used in this study. The study subjects were limited to adults aged ≥ 19 years who resided in the following eight cities: Seoul, Incheon, Gwangju, Jeju-do, Busan, Ulsan, Daegu, and Daejeon. The selection of these cities was based on their high population density and geographical dispersion. These cities also provide distinct contexts due to each city’s layout, density, socioeconomic conditions, and green space policies. The outline of each city is as follows:

- Seoul: Seoul is the capital and the largest city with high population density and limited green space relative to its population size. Efforts have been made to increase green space per capita such as through projects like the Seoul Forest and Cheonggyecheon Stream restoration. However, disparities in green space accessibility persist between neighborhoods within Seoul.
- Incheon: Incheon, located near Seoul, is a growing port city with more recent development projects aimed at creating eco-friendly spaces, especially around Songdo International Business District. Incheon’s urban planning has incorporated green spaces into new developments, aiming to counterbalance its industrialized zones.
- Gwangju: Known for its history and cultural significance, Gwangju has moderate population density and is surrounded by scenic, mountainous terrain. The city emphasizes community parks and greenways, offering residents access to outdoor recreational spaces.

- Jeju-do: As an island province, Jeju-do has a unique landscape with abundant green space, including volcanic mountains, coastal parks, and trails. With focus on tourism and eco-friendly development, Jeju-do’s residents and visitors have high access to natural spaces.

Busan: South Korea’s second-largest city, Busan, is a coastal metropolis with high population density in some districts, but also considerable access to green spaces like Haeundae Beach and Geumjeonsan Mountain. Busan’s green spaces are concentrated in certain areas, leading to accessibility gaps. However, the coastal and hilly landscape encourages outdoor activities.

 Ulsan: An industrial city known for its automotive and petrochemical industries, Ulsan has a higher concentration of industrial zones, limiting green space in some urban areas. However, the city has made efforts to increase green areas and parks, particularly along the Taehwagang River.

- Daegu: Situated in an inland valley, Daegu is one of the major cities and has faced challenges in green space provision due to its compact layout. The city has been working to increase its green areas, but it has limited urban green space, especially in central districts.

Daejeon: Known as the hub for science and technology, Daejeon has invested in green infrastructure with spaces like the Expo Science Park and Gyeryongsan National Park. Its relatively balanced layout and moderate density allow for a higher per capita green space than other cities.

The data of 1,014,606 subjects who participated in the KCHS from 2008 to 2021 were initially considered for this study. Subjects that responded “refused to respond” or “do not know” to any of the survey questions were excluded from the study, as were those with outlier values (BMI: < 10 or > 50 kg/m^2^). Consequently, the final analysis encompassed a total of 674,962 subjects.

The study design and data analysis protocol were reviewed and approved by the Institutional Review Board of the National Medical Center (IRB No. 2023-06-069; IRB examination exemption approval 2023-06-22).

### BMI measurement

**Fig. 1:**
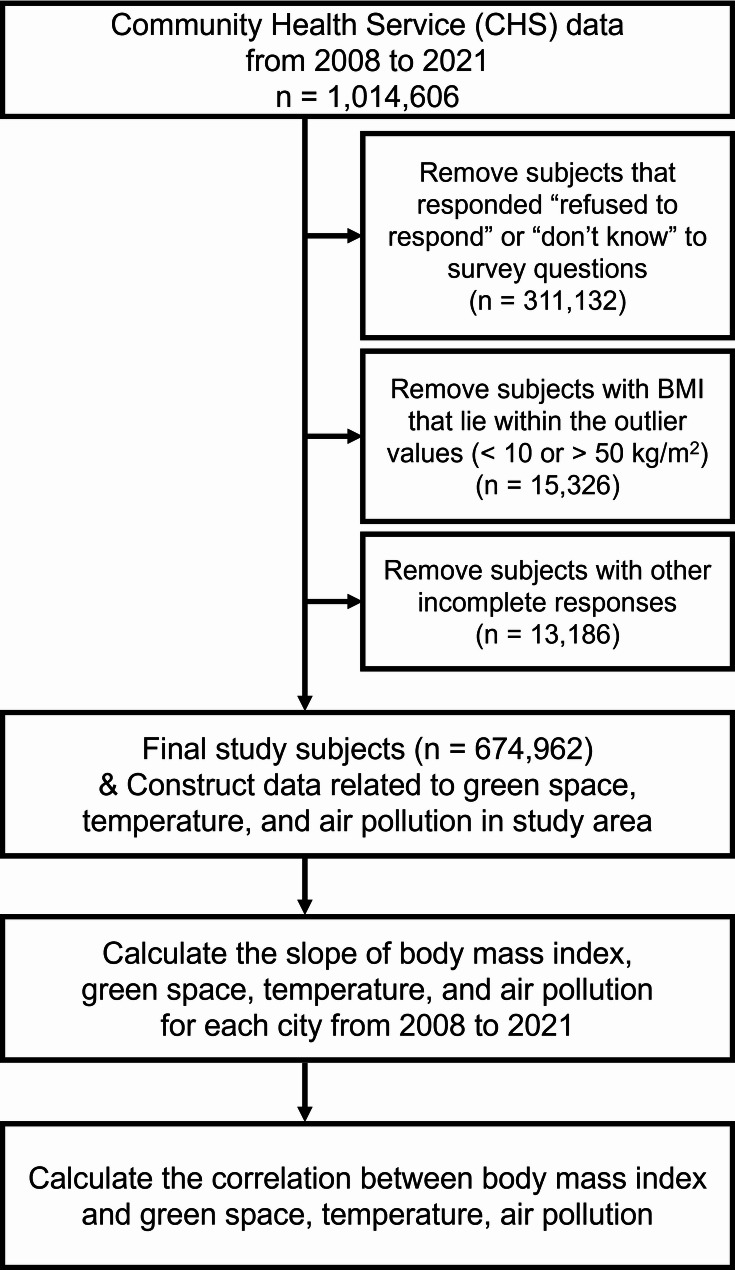
Schematic illustration of the study subject selection process and deriving the result for analysis. This illustration portrays the process of the study subject selection from the Community Health Service data. Abbreviations: *n*, number of subjects; BMI, body mass index

BMI was calculated using the subject’s weight in kilograms divided by the square of the subject’s height in meters. As the weight and height from 2008 to 2017 were self-reported, BMI values that were < 10 kg/m^2^ or > 50 kg/m^2^ were excluded to remove data that were unlikely to be valid. Exclusion of outlier BMI values was stopped after 2018 when weight and height were measured directly during face- to-face interviews (Fig. 1).

Following the calculation of BMI, the study subjects were categorised in accordance with the Asia-Pacific classification of obesity for Asian adults as follows: normal (BMI: 18.5–22.9 kg/m^2^), overweight (BMI: 23–24.9 kg/m^2^), and obesity class 1–3 (class 1: BMI 25–29.9 kg/m^2^; class 2: BMI: 30–34.9 kg/m^2^; class 3: BMI: ≥ 35 kg/m^2^).

### Measurement of green space

In this study, green space is represented as park data, which were acquired from the Korean Statistical Information Service from 2008 to 2021. The types of parks included urban natural parks, national urban parks, small parks, children’s parks, neighborhood parks, historical parks, cultural parks, waterfront parks, cemetery parks, sports parks, urban agricultural parks, disaster prevention parks, and parks prescribed by the ordinance. This study exclusively used park data to represent green space because parks were the only type of green space data consistently available across all eight cities included in our analysis. All of these park areas in each city were summed and used in the form of square meters (m^2^).

### Measurement of ambient temperature

**Fig. 2:**
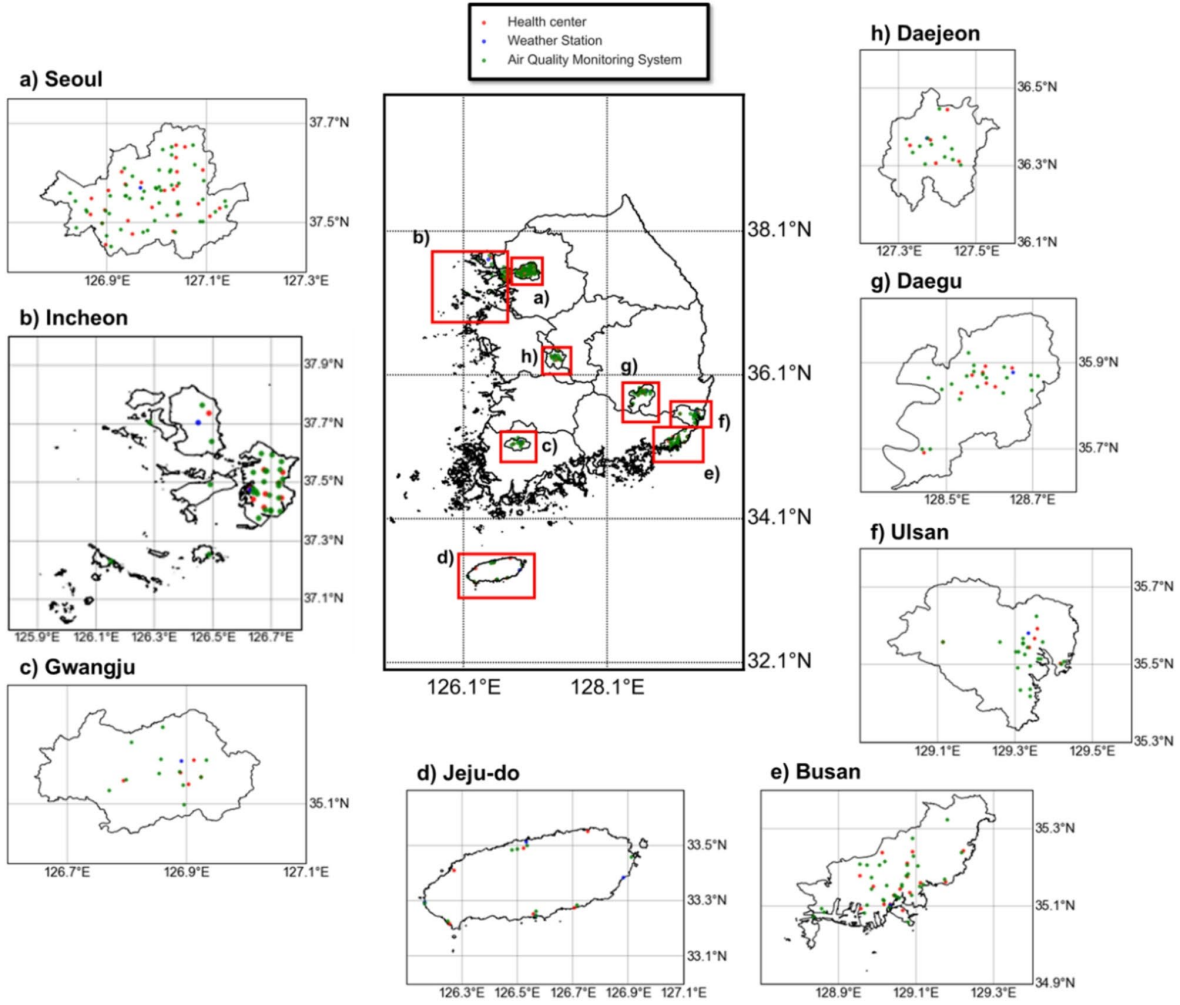
Geographical location of health centers, weather stations, and air quality monitoring system (Air Korea) stations in the study area

Observed data on the average daily maximum ambient temperature in degrees Celsius were collected from Automated Synoptic Observation System (ASOS) weather stations run by the Korea Meteorological Administration. Our data were collected from 13 of the 96 ASOS weather stations nationally, all of which were located in the eight cities included in the study (Fig. 2).

### Measurement of air pollution

In this study, air pollution was defined as particulate matter and air pollutants that were collected by Air Korea stations that are run by the Korea Environment Corporation (Fig. 2). The Air Korea stations provide real-time data, offering hourly to annual data on diverse forms of air pollutants. The particulate matter included in this study were those with aerodynamic diameters ≤ 10 μm (PM10) and diameters ≤ 2.5 μm (PM2.5). PM10 data were available from 2008 to 2021, but PM2.5 data were only available from 2015 to 2021 due to late initiation of observation by the Korea Environment Corporation. The air pollutants included in this study were sulfur dioxide (SO_2_), nitrogen dioxide (NO_2_), ozone (O_3_), and carbon monoxide (CO). These air-pollutant data were available from 2008 to 2021. Among these variables, only PM10, PM2.5, and O_3_ had grading standards that categorized the predicted concentration into ‘good’, ‘moderate’, ‘unhealthy’, and ‘very unhealthy’.

### Covariates

Demographic characteristics, such as socioeconomic status and health behaviors, of the study subjects were included in the study. Specifically, age (19 ∼ 44, 45 ∼ 64, and ≥ 64 years), sex (male, female), smoking (non-smoker, ex-smoker, and current smoker), alcohol drinking (drinker and non-drinker), monthly household income (≤ 1,000,000, 1,000,000 ∼ 2,000,000, 2,000,000 ∼ 3,000,000, 3,000,000 ∼ 4,000,000, 4,000,000 ∼ 5,000,000, and ≥ 5,000,000 South Korean Won), education level (elementary school or lower, middle school, high school, and college or higher), marital status (married, never married, and separated/divorced/widowed), physical activity (yes or no), and stress (yes or no) were included in the study.

### Statistical analysis

Weighted frequency analysis was conducted with individual and household weights applied to individual variables to examine the demographic characteristics of the study subjects [7]. The Pearson correlation coefficient was used to calculate the correlation between green space, ambient temperature, and air pollution with obesity. *P*-values < 0.05 were considered statistically significant [8]. Our study, with only a few nonparametric variables that did not follow normal distribution, utilized robust regression, specifically the Theil-Sen estimator, to easily understand the direction of the trend during the study period. Compared to simple linear regression, this estimator can be computed efficiently and is insensitive to outliers [13]. All data processing and analyses were performed using Python (v3.10).

## Results

### Characteristics of the study subjects

Descriptive statistics of the study subjects are presented in Table 1. Among the 674,962 study subjects, 56.88% were female, with a mean age of 52.67 years. The mean age of individuals was relatively consistent across all BMI classes, but the obesity class 2 and 3 groups had the lowest mean age. The proportion of female study subjects was slightly higher in all the BMI classes, but the proportion of males was higher in the obesity class 1 group (51.42%).

**Table 1:** Characteristics of the study subjects based on body mass index

Variable	Total (*n* = 674,962)	Normal (*n* = 323,126)	Overweight (*n* = 168,654)	Class 1 (*n* = 163,322)	Class 2 (*n* = 17,760)	Class 3 (*n* = 2,100)
Age, years, mean (SD)	52.67 (16.22)	51.13 (17.21)	54.77 (14.88)	54.04 (15.02)	49.30 (16.56)	43.57 (17.48)
19–44	33.42 (7.48)	32.86 (7.65)	34.19 (7.34)	34.21 (7.13)	32.70 (7.37)	31.61 (7.87)
45–64	55.29 (5.69)	55.16 (5.70)	55.81 (5.62)	55.12 (5.67)	54.13 (5.83)	53.48 (6.21)
≥ 65	73.95 (6.57)	74.95 (7.01)	73.37 (6.22)	72.98 (5.94)	72.87 (5.99)	72.69 (5.67)
Sex, No. (%)						
Male	291,036 (43.12%)	115,255 (35.67%)	82,208 (48.74%)	83,982 (51.42%)	8,607 (48.46%)	984 (46.86%)
Female	383,926 (56.88%)	207,871 (64.33%)	86,446 (51.26%)	79,340 (48.58%)	9,153 (51.54%)	1,116 (53.14%)
BMI, kg/m^2^, mean (SD)	23.35 (3.20)	20.73 (1.65)	23.95 (0.58)	26.73 (1.28)	31.56 (1.27)	37.53 (2.63)
Smoking, No. (%)						
Non-smoker	428,195 (63.44%)	220,769 (68.32%)	101,484 (60.17%)	94,038 (57.58%)	10,546 (59.38%)	1,358 (64.67%)
Ex-smoker	111,983 (16.59%)	41,490 (12.84%)	32,996 (19.56%)	34,218 (20.95%)	3,020 (17.00%)	259 (12.33%)
Current smoker	134,784 (19.97%)	60,867 (18.84%)	34,174 (20.26%)	35,066 (21.47%)	4,194 (23.61%)	483 (23.00%)
Alcohol drinking, No. (%)						
Drinker	543,721 (80.56%)	257,924 (79.82%)	136,533 (80.95%)	133,135 (81.52%)	14,420 (81.19%)	1,709 (81.38%)
Non-drinker	131,241 (19.44%)	65,202 (20.18%)	32,121 (19.05%)	30,187 (18.48%)	3,340 (18.81%)	391 (18.62%)
Monthly household income, No. (%)	308.53 (281.97)	308.47 (278.17)	308.17 (292.12)	309.50 (282.79)	304.50 (242.23)	305.54 (281.56)
≤ ₩1,000,000	99,440 (14.73%)	48,270 (14.94%)	24,561 (14.56%)	23,622 (14.46%)	2,671 (15.04%)	316 (15.05%)
₩1,000,000 ∼ 2,000,000	125,609 (18.61%)	59,197 (18.32%)	32,200 (19.09%)	30,564 (18.71%)	3,274 (18.43%)	374 (17.81%)
₩2,000,000 ∼ 3,000,000	132,875 (19.69%)	63,599 (19.68%)	33,139 (19.65%)	32,229 (19.73%)	3,470 (19.54%)	438 (20.86%)
₩3,000,000 ∼ 4,000,000	114,303 (16.93%)	54,740 (16.94%)	28,459 (16.87%)	27,638 (16.92%)	3,122 (17.58%)	344 (16.38%)
₩4,000,000 ∼ 5,000,000	73,813 (10.94%)	35,448 (10.97%)	18,306 (10.85%)	17,899 (10.96%)	1,917 (10.79%)	243 (11.57%)
≥ ₩5,000,000 or less	128,922 (19.10%)	61,872 (19.15%)	31,989 (18.97%)	31,370 (19.21%)	3,306 (18.61%)	385 (18.33%)
Education level, No. (%)						
Elementary school or lower	133,865 (19.83%)	60,037 (18.59%)	34,555 (20.49%)	35,343 (21.64%)	3,567 (20.08%)	363 (17.29%)
Middle school	98,216 (14.55%)	41,810 (12.94%)	27,986 (16.59%)	25,929 (15.88%)	2,298 (12.94%)	193 (9.19%)
High school	276,279 (40.93%)	131,809 (40.79%)	70,317 (41.69%)	66,082 (40.46%)	7,161 (40.32%)	910 (43.33%)
College or higher	166,602 (24.68%)	89,470 (27.69%)	35,796 (21.22%)	35,968 (22.02%)	4,734 (26.66%)	634 (30.19%)
Marital status, No. (%)						
Married	579,070 (85.79%)	275,051 (85.12%)	146,894 (87.10%)	140,461 (86.09%)	14,821 (83.45%)	1,703 (81.10%)
Never married	28,696 (4.25%)	14,897 (4.61%)	6,379 (3.78%)	6,561 (4.02%)	778 (4.38%)	81 (3.86%)
Separated/divorced/widowed	67,196 (9.96%)	33,178 (10.27%)	15,381 (9.12%)	16,160 (9.89%)	2,161 (12.17%)	316 (15.05%)
Physical activity, No. (%)						
Yes	491,695 (72.85%)	236,895 (73.31%)	124,108 (73.59%)	117,246 (71.79%)	12,085 (68.05%)	1,361 (64.81%)
No	183,267 (27.15%)	86,231 (26.69%)	44,546 (26.41%)	46,076 (28.21%)	5,675 (31.95%)	739 (35.19%)
Stress, No. (%)						
No	136,331 (20.20%)	61,753 (19.11%)	36,051 (21.38%)	34,082 (21.31%)	3,377 (19.01%)	348 (16.57%)
Yes	538,631 (79.80%)	261,373 (80.89%)	132,603 (78.62%)	128,520 (78.69%)	14,383 (80.99%)	1,752 (83.43%)

Abbreviations: *n*, number of subjects; BMI, body mass index

Compared to the normal weight group, the subjects who were overweight or obese all smoke or drank alcohol more. In contrast, marital status and socioeconomic status, such as household income and education, did not seem to be related to BMI. The proportion of subjects who reported being engaged in physical activity was highest in the overweight group, followed by the normal weight group. The percentage decreased as BMI increased, with the obesity class 1 group at 71.79%, class 2 group at 68.05%, and class 3 group at 64.81%. The difference in the percentage of study subjects who reported stress was highest in the obesity class 3 group (66.86%).

### Trends in body mass index, green space, ambient temperature, and air pollution

The BMI of the study population in eight cities and the total area of these cities was observed from 2008 to 2021 (Table 2). For the normal weight group, the BMI increased in all of the cities and in the total region of the cities. This increasing trend in BMI was also observed in the obesity class 1 and 2 groups. The overweight and obesity class 3 group did not show a clear trend in BMI and did not show a decreasing trend in BMI in any city.

**Table 2:** Trend of body mass index from 2008 to 2021

	Normal (*n* = 323,126)			Overweight (*n* = 168,654)			Class 1 (*n* = 163,322)			Class 2 (*n* = 17,760)			Class 3 (*n* = 2,100)		
	Trend	*p*-value	Slope	Trend	*p*-value	Slope	Trend	*p*-value	Slope	Trend	*p*-value	Slope	Trend	*p*-value	Slope
Seoul	Increasing	< 0.001	0.011	No trend	0.274	< 0.001	Increasing	0.001	0.013	No trend	0.063	0.008	No trend	0.324	0.038
Incheon	Increasing	< 0.001	0.018	No trend	0.155	0.003	Increasing	< 0.001	0.015	Increasing	0.016	0.018	No trend	0.274	0.051
Gwangju	Increasing	0.004	0.019	No trend	0.661	< 0.001	No trend	0.080	0.015	Increasing	0.029	0.020	No trend	0.743	0.037
Jeju-do	Increasing	0.001	0.016	No trend	0.584	0.001	Increasing	< 0.001	0.015	Increasing	0.016	0.026	No trend	0.511	0.052
Busan	Increasing	< 0.001	0.011	No trend	0.324	0.001	Increasing	< 0.001	0.012	Increasing	0.004	0.016	No trend	0.443	0.013
Ulsan	Increasing	0.009	0.013	Increasing	0.037	0.004	Increasing	0.009	0.019	No trend	0.443	0.013	No trend	0.913	0.013
Daegu	Increasing	0.001	0.013	No trend	0.827	–0.0001	Increasing	0.002	0.015	No trend	0.913	–0.001	No trend	0.661	–0.023
Daejeon	Increasing	0.002	0.015	No trend	0.584	< 0.001	Increasing	0.012	0.016	Increasing	0.021	0.020	Increasing	0.037	0.150
Total	Increasing	< 0.001	0.013	No trend	0.101	0.002	Increasing	< 0.001	0.013	Increasing	0.003	0.014	Increasing	0.009	0.056

The trend in the table is an interpretation of the slope, which is calculated via Theil-Sen regression. Abbreviations: *n*, number of subjects

**Fig. 3:**
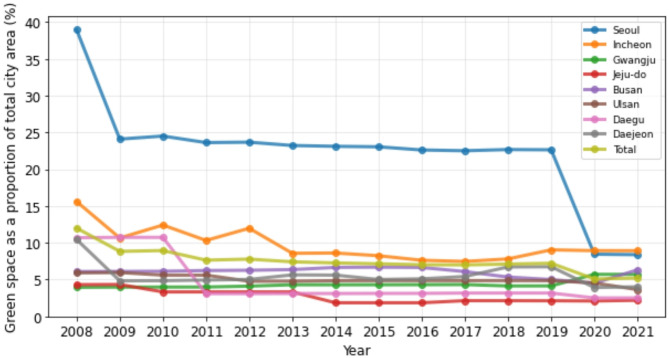
Green space as a proportion of total city area (%) from 2008 to 2021

**Fig. 4:**
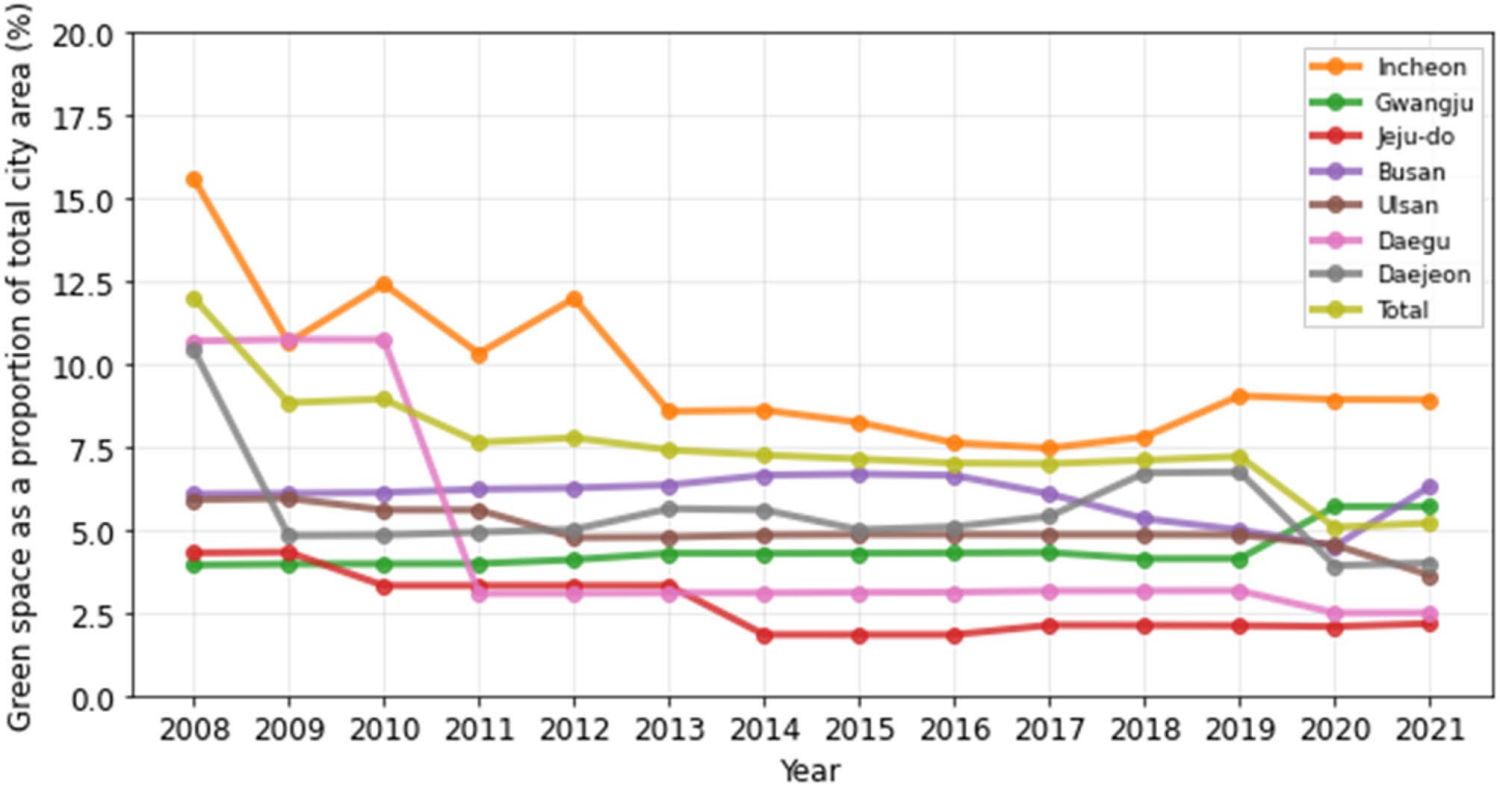
Green space as a proportion of total city area (%) from 2008 to 2021. Seoul, one of the study areas, has been excluded from the graph to allow more clearer representation of the changes in green space across the remaining study areas over the study period

Table 3 shows the percentage of green space as a proportion of the total city area for each of the eight cities and total region for cities from 2008 to 2021. Overall, the trend of green space showed a gradual decline in percentage from 2008 to 2021. In 2008, the total percentage of green space in the study area was 11.98%, which decreased to 5.19% in 2021. This trend was consistent across most of the cities; Seoul, the capital city, had the highest percentage of green space in 2008, at 38.96%, which decreased to 8.37% in 2021. Gwangju, a smaller city compared to Seoul, initially had a relatively low percentage of green space in 2008, at 3.93%, but was the only city that showed a steady increase over the years, reaching 5.70% in 2021. The trends of green space in the rest of the cities are presented in Figs. 3 and 4.

**Table 3:** Green space as a proportion of total city area (%) from 2008 to 2021

	2008	2009	2010	2011	2012	2013	2014	2015	2016	2017	2018	2019	2020	2021
Seoul	38.96	24.09	24.50	23.61	23.67	23.22	23.11	23.04	22.61	22.51	22.67	22.63	8.44	8.37
Incheon	15.59	10.64	12.40	10.31	11.97	8.56	8.60	8.23	7.61	7.46	7.79	9.03	8.92	8.91
Gwangju	3.93	3.96	3.97	3.97	4.09	4.28	4.28	4.29	4.30	4.31	4.12	4.12	5.69	5.70
Jeju-do	4.30	4.32	3.31	3.31	3.31	3.31	1.83	1.83	1.83	2.12	2.12	2.11	2.08	2.17
Busan	6.07	6.09	6.12	6.21	6.25	6.34	6.64	6.68	6.63	6.07	5.33	4.99	4.50	6.31
Ulsan	5.90	5.94	5.59	5.59	4.76	4.77	4.84	4.85	4.85	4.85	4.85	4.84	4.53	3.62
Daegu	10.68	10.73	10.72	3.08	3.08	3.09	3.09	3.10	3.11	3.15	3.15	3.16	2.49	2.49
Daejeon	10.44	4.82	4.84	4.92	5.00	5.63	5.59	4.99	5.09	5.40	6.71	6.73	3.91	4.00
Total	11.98	8.82	8.93	7.63	7.77	7.40	7.25	7.13	7.00	6.98	7.09	7.20	5.07	5.19

Except for Gwangju, most of the cities exhibited a decreasing trend in green space and an increasing trend in ambient temperature during the study period from 2008 to 2021 (Table 4). This pattern is evident in Seoul, Incheon, Jeju-do, and in the overall total region, which may imply that as the green space in cities decreases, the temperature increases. Conversely, in Gwangju, where the trend for green space is increasing, there is no clear trend in temperature, which may suggest that the growth of green space has helped to alleviate the rise in temperature.

**Table 4:** Trend of green space and average daily maximum ambient temperature from 2008 to 2021

		Trend	*p*-value	Slope
Seoul	Green space	Decreasing	< 0.001	–0.003
	Temperature	Increasing	0.036	0.150
Incheon	Green space	Decreasing	0.021	–0.003
	Temperature	Increasing	0.037	0.081
Gwangju	Green space	Increasing	< 0.001	< 0.001
	Temperature	No trend	0.095	0.067
Jeju-do	Green space	Decreasing	0.017	–0.002
	Temperature	Increasing	0.007	0.069
Busan	Green space	No trend	0.827	> − 0.001
	Temperature	Increasing	0.008	0.075
Ulsan	Green space	Decreasing	0.006	–0.001
	Temperature	No trend	0.700	0.011
Daegu	Green space	No trend	0.511	–0.001
	Temperature	No trend	0.544	0.020
Daejeon	Green space	No trend	0.827	< 0.001
	Temperature	Increasing	0.003	0.137
Total	Green space	Decreasing	< 0.001	–0.278
	Temperature	Increasing	0.006	0.072

The trend in the table is an interpretation of the slope, which is calculated via Theil-Sen regression

Concentrations of air pollutants (i.e., PM10, NO_2_, SO_2_, and CO) indicated a slightly decreasing trend in all cities from 2008 to 2021 (Table 5). In the case of PM10, this tendency in all the cities was within the ‘moderate’ grade according to the established criteria (Table 6). Jeju-do, which was categorized as ‘moderate’ until 2016, as well as showed a drastic decline in PM10 in 2017 and improved to ‘good’ grade. Other cities, such as Incheon, Gwangju, Busan, and Ulsan, also reached ‘good’ grade in 2020.

**Table 5:** Trend analysis of air pollution from 2008 to 2021

		Trend	*p*-value	Slope
Seoul	PM10	Decreasing	0.003	–0.858
	PM2.5	Increasing	0.019	1.734
	NO_2_	Decreasing	0.006	–0.0008
	SO_2_	Decreasing	0.012	–0.0002
	O_3_	Increasing	< 0.001	0.0006
	CO	Decreasing	< 0.001	–0.0072
Incheon	PM10	Decreasing	< 0.001	–1.011
	PM2.5	Increasing	0.035	1.269
	NO_2_	Decreasing	< 0.001	–0.0008
	SO_2_	Decreasing	0.001	–0.0002
	O_3_	Increasing	< 0.001	0.0005
	CO	Decreasing	< 0.001	–0.0087
Gwangju	PM10	Decreasing	< 0.001	–1.234
	PM2.5	No trend	0.108	1.163
	NO_2_	Decreasing	< 0.001	–0.0008
	SO_2_	Decreasing	0.001	–0.0002
	O_3_	Increasing	< 0.001	0.0005
	CO	Decreasing	< 0.001	–0.0094
Jeju-do	PM10	Decreasing	< 0.001	–1.286
	PM2.5	No trend	0.386	1.504
	NO_2_	Decreasing	< 0.001	–0.0007
	SO_2_	Decreasing	< 0.001	–0.0002
	O_3_	Increasing	< 0.001	0.0005
	CO	Decreasing	< 0.001	–0.0107
Busan	PM10	Decreasing	0.003	–1.193
	PM2.5	No trend	0.536	0.512
	NO_2_	Decreasing	0.003	–0.0007
	SO_2_	Decreasing	0.003	–0.0002
	O_3_	Increasing	< 0.001	0.0006
	CO	Decreasing	< 0.001	–0.0098
Ulsan	PM10	Decreasing	< 0.001	–1.281
	PM2.5	No trend	0.174	1.673
	NO_2_	Decreasing	< 0.001	–0.0007
	SO_2_	Decreasing	< 0.001	–0.0002
	O_3_	Increasing	< 0.001	0.0005
	CO	Decreasing	< 0.001	–0.0093
Daegu	PM10	Decreasing	< 0.001	–1.145
	PM2.5	No trend	0.266	0.759
	NO_2_	Decreasing	< 0.001	–0.0007
	SO_2_	Decreasing	0.001	–0.0002
	O_3_	Increasing	< 0.001	0.0005
	CO	Decreasing	< 0.001	–0.0104
Daejeon	PM10	Decreasing	< 0.001	–1.084
	PM2.5	No trend	0.174	1.310
	NO_2_	Decreasing	< 0.001	–0.0008
	SO_2_	Decreasing	< 0.001	–0.0002
	O_3_	Increasing	< 0.001	0.0005
	CO	Decreasing	< 0.001	–0.0097
Total	PM10	Decreasing	< 0.001	–1.132
	PM2.5	No trend	0.266	1.352
	NO_2_	Decreasing	< 0.001	–0.0007
	SO_2_	Decreasing	0.001	–0.0002
	O_3_	Increasing	< 0.001	0.0005
	CO	Decreasing	< 0.001	–0.0096

The trend in the table is an interpretation of the slope, which is calculated via Theil-Sen regression. Abbreviation: PM, particulate matter

**Table 6:** Air pollutant grades according to concentration

Forecast Category		Grades			
		Good	Moderate	Unhealthy	Very Unhealthy
Predicted Concentration (μg/m^3^, day)	PM10	0∼30	31∼80	81∼150	> 151
	PM2.5	0∼15	16∼35	36∼75	> 76
Predicted Concentration (ppm, 1 h)	O_3_	0∼0.030	0.031∼0.90	0.091∼0.150	> 0.151

Data and guidelines are from the Korea Environment Corporation, which provides the Air Korea stations that measures the air pollutants in the study area. Abbreviation: PM, particulate matter

The levels of NO_2_, CO, and SO_2_ have gradually decreased over the years, though with some fluctuations in between. These pollutants (i.e., NO_2_, CO, and SO_2_) unlike PM10, PM2.5, and O_3_, do not have a criterion to evaluate their severity, but Seoul showed the highest concentration of pollutants for both NO_2_ and CO, while Jeju-do showed the lowest levels of NO_2_ and CO.

Moreover, O_3_ showed an increasing trend of all the cities, with the most significant increase shown in the two largest cities in South Korea–Seoul and Busan (slope = 0.0006). The O_3_ was graded according to measured concentration (ppm) per hour (Table 6). Although the most significant increase of O_3_ measured in other cities, only Jeju-do had the highest levels of O_3_. Furthermore, Jeju-do maintained the ‘moderate’ grade throughout the study period, while all of the other cities were graded as ‘good’.

PM2.5 also increased throughout the study years, but this increasing trend was very limited and was statistically significant in only two cities– Seoul and Incheon. The trends in the remaining cities during the study period were not significant. Nevertheless, the highest levels of PM2.5 in 2015 were noted in Incheon, Busan, and Gwangju, all of which were graded as ‘moderate’ based on the predicted concentration during the day (Table 6). By 2021, only Daejeon remained in the ‘good’ grade for PM2.5. The PM2.5 grades in the other cities fluctuated over time, with none of them maintaining their initial ‘good’ grade.

### Green space and body mass index

Table 7 presents the correlation between green space and BMI. Among the eight cities, only Seoul, Incheon, and Ulsan showed statistically significant negative correlations in the normal weight group, indicating that a decrease in green space was associated with an increase in BMI. However, in the normal weight group of Gwangju, a positive correlation was observed, indicating that an increase in green space was associated with an increase in BMI. The remaining cities showed no statistically significant correlations. In the obesity class 1–2 groups, Jeju-do consistently showed a negative association, indicating that a decrease in green space was associated with an increase in BMI. In contrast, Daegu showed a positive correlation in the obesity class 2 group, indicating that a decrease in green space was associated with a decrease in BMI. None of the cities showed any significant correlation between the overweight and obesity class 3 groups. In the total region of the cities, only the normal weight group showed a meaningful negative correlation.

**Table 7:** Correlation between green space and body mass index

	Normal (*n* = 323,126)		Overweight (*n* = 168,654)		Class 1 (*n* = 163,322)		Class 2 (*n* = 17,760)		Class 3 (*n* = 2,100)	
	Corr.	*p*-value	Corr.	*p*-value	Corr.	*p*-value	Corr.	*p*-value	Corr.	*p*-value
Seoul	–0.81	< 0.001	–0.37	0.198	–0.47	0.090	–0.50	0.066	–0.14	0.633
Incheon	–0.65	0.012	–0.36	0.202	–0.64	0.014	–0.38	0.186	–0.46	0.099
Gwangju	0.61	0.020	0.06	0.840	0.32	0.912	0.26	0.371	0.23	0.427
Jeju-do	–0.50	0.066	–0.02	0.952	–0.72	0.004	–0.57	0.032	–0.19	0.507
Busan	–0.31	0.280	0.40	0.152	–0.42	0.131	–0.11	0.696	–0.01	0.975
Ulsan	–0.71	0.005	–0.48	0.084	–0.44	0.112	–0.21	0.474	0.16	0.581
Daegu	–0.44	0.120	0.17	0.550	–0.42	0.137	0.59	0.026	–0.04	0.901
Daejeon	–0.33	0.250	–0.10	0.737	0.09	0.751	–0.16	0.585	–0.15	0.597
Total	–0.68	0.007	–0.34	0.234	–0.52	0.055	–0.47	0.089	–0.35	0.219

Abbreviation: *n*, number of subjects; Corr, correlation

### Ambient temperature and body mass index

In general, there was a positive correlation between ambient temperature and BMI for most cities in the normal weight group and obesity class 1 group, as shown in Table 8. However, in the overweight group, only Busan, Ulsan, and Daegu showed significant positive correlations. The total area of the cities revealed positive correlations between ambient temperature and BMI in the normal weight, overweight, and obesity class 1 group. Conversely, obesity classes 2 and 3 showed no significant correlation between ambient temperature and BMI in the total area of the cities.

**Table 8:** Correlation between average daily maximum ambient temperature and body mass index

	Normal (*n* = 323,126)		Overweight (*n* = 168,654)		Class 1 (*n* = 163,322)		Class 2 (*n* = 17,760)		Class 3 (*n* = 2,100)	
	Corr.	*p*-value	Corr.	*p*-value	Corr.	*p*-value	Corr.	*p*-value	Corr.	*p*-value
Seoul	0.62	0.019	0.19	0.516	0.51	0.063	0.47	0.094	0.23	0.431
Incheon	0.71	0.004	0.45	0.102	0.59	0.025	0.29	0.314	–0.08	0.780
Gwangju	0.55	0.404	0.16	0.579	0.64	0.013	0.19	0.511	–0.22	0.441
Jeju-do	0.73	0.003	0.41	0.144	0.71	0.004	0.38	0.19	0.41	0.145
Busan	0.77	0.001	0.72	0.003	0.68	0.007	0.47	0.087	0.20	0.498
Ulsan	0.60	0.024	0.66	0.010	0.35	0.213	0.26	0.365	–0.21	0.473
Daegu	0.64	0.013	0.56	0.036	0.53	0.050	0.01	0.983	–0.26	0.365
Daejeon	0.87	< 0.000	0.18	0.532	0.67	0.008	0.45	0.103	0.60	0.022
Total	0.89	< 0.000	0.681	0.007	0.72	0.004	0.41	0.143	0.49	0.078

Abbreviation: *n*, number of subjects; Corr, correlation

### Air pollution and body mass index

Looking at the correlation between air pollution and BMI, most of the cities showed a negative correlation between PM10, NO_2_, and CO with BMI– these findings were seen in the normal weight group and in all obesity classes. However, the correlation between O_3_ and BMI was not significant in most cities, except in the total region, where it showed a positive correlation, particularly in the normal weight and overweight groups. These findings are presented in Table 9.

**Table 9:** Correlation between air pollution and body mass index

		Normal (*n* = 323,126)		Overweight (*n* = 168,654)		Class 1 (*n* = 163,322)		Class 2 (*n* = 17,760)		Class 3 (*n* = 2,100)	
		Corr.	*p*-value	Corr.	*p*-value	Corr.	*p*-value	Corr.	*p*-value	Corr.	*p*-value
Seoul	PM10	–0.60	0.023	–0.32	0.272	–0.66	0.01	–0.37	0.194	–0.02	0.959
	PM2.5	0.27	0.512	–0.02	0.966	0.13	0.754	0.75	0.034	0.34	0.405
	NO_2_	–0.69	0.006	–0.10	0.741	–0.61	0.02	–0.39	0.169	–0.37	0.197
	SO_2_	–0.70	0.005	–0.14	0.63	–0.44	0.115	–0.46	0.101	–0.16	0.581
	O_3_	0.79	0.001	0.07	0.813	0.59	0.026	0.68	0.008	0.01	0.961
	CO	0.14	0.642	–0.07	0.804	0.22	0.442	0.26	0.362	0.34	0.23
Incheon	PM10	–0.62	0.019	–0.06	0.848	–0.53	0.05	–0.73	0.003	–0.21	0.472
	PM2.5	0.36	0.382	0.31	0.451	0.75	0.034	0.59	0.126	0.35	0.395
	NO_2_	–0.65	0.012	–0.17	0.564	–0.58	0.031	–0.67	0.009	–0.18	0.528
	SO_2_	–0.73	0.003	–0.19	0.514	–0.62	0.018	–0.65	0.011	–0.10	0.726
	O_3_	0.69	0.006	0.63	0.016	0.60	0.024	0.24	0.414	0.39	0.169
	CO	–0.41	0.15	–0.05	0.871	–0.43	0.126	–0.61	0.021	–0.21	0.48
Gwangju	PM10	–0.69	0.006	0.07	0.799	–0.38	0.175	–0.48	0.08	–0.23	0.422
	PM2.5	0.46	0.247	0.08	0.842	0.71	0.048	–0.09	0.831	0.47	0.244
	NO_2_	–0.65	0.011	–0.07	0.821	–0.47	0.091	–0.57	0.033	–0.18	0.547
	SO_2_	–0.45	0.11	–0.03	0.908	–0.74	0.003	–0.59	0.027	–0.01	0.966
	O_3_	0.43	0.124	0.11	0.71	0.16	0.591	0.24	0.408	0.15	0.616
	CO	–0.55	0.041	–0.06	0.831	–0.56	0.039	–0.62	0.018	–0.15	0.62
Jeju-do	PM10	–0.38	0.181	–0.16	0.575	–0.74	0.002	–0.43	0.123	–0.09	0.752
	PM2.5	0.46	0.25	0.13	0.751	0.60	0.117	–0.10	0.816	–0.14	0.747
	NO_2_	–0.23	0.419	–0.01	0.982	–0.54	0.046	–0.61	0.02	0.14	0.636
	SO_2_	–0.53	0.05	–0.01	0.98	–0.70	0.005	–0.68	0.008	–0.12	0.688
	O_3_	–0.02	0.947	0.26	0.378	–0.34	0.231	–0.10	0.731	–0.04	0.885
	CO	–0.65	0.012	–0.19	0.506	–0.85	< 0.000	–0.31	0.275	–0.19	0.519
Busan	PM10	–0.70	0.005	0.03	0.914	–0.752	0.002	–0.55	0.044	–0.20	0.497
	PM2.5	0.32	0.443	0.20	0.635	0.44	0.272	0.08	0.851	0.31	0.455
	NO_2_	–0.60	0.022	0.03	0.922	–0.58	0.031	–0.56	0.037	0.09	0.754
	SO_2_	–0.73	0.003	0.06	0.843	–0.68	0.007	–0.61	0.02	0.01	0.967
	O_3_	0.86	< 0.000	0.46	0.096	0.58	0.029	0.51	0.06	0.23	0.433
	CO	–0.61	0.02	–0.16	0.592	–0.68	0.008	–0.60	0.022	–0.17	0.553
Ulsan	PM10	–0.63	0.016	–0.41	0.145	–0.62	0.018	–0.22	0.449	–0.13	0.651
	PM2.5	0.22	0.609	0.02	0.967	0.50	0.211	0.03	0.946	0.39	0.338
	NO_2_	–0.44	0.113	–0.22	0.449	–0.52	0.056	–0.05	0.853	–0.29	0.318
	SO_2_	–0.59	0.028	–0.41	0.146	–0.58	0.031	–0.11	0.716	–0.28	0.332
	O_3_	0.87	< 0.000	0.67	0.009	0.60	0.023	0.32	0.262	–0.15	0.605
	CO	–0.17	0.57	–0.09	0.767	0.18	0.529	–0.11	0.696	–0.02	0.955
Daegu	PM10	–0.73	0.003	0.07	0.799	–0.74	0.003	0.49	0.072	0.03	0.911
	PM2.5	0.88	0.003	–0.07	0.863	0.23	0.576	0.38	0.356	0.20	0.627
	NO_2_	–0.73	0.003	0.10	0.737	–0.66	0.011	0.39	0.174	–0.04	0.89
	SO_2_	–0.81	< 0.000	–0.02	0.959	–0.74	0.002	0.42	0.136	0.04	0.885
	O_3_	0.58	0.03	0.21	0.47	0.62	0.018	–0.20	0.485	0.14	0.645
	CO	–0.57	0.032	0.13	0.669	–0.44	0.117	0.56	0.037	0.12	0.685
Daejeon	PM10	–0.38	0.184	0.23	0.42	–0.18	0.523	–0.35	0.215	–0.37	0.195
	PM2.5	0.01	0.986	0.32	0.441	0.693	0.057	0.83	0.011	0.27	0.517
	NO_2_	–0.56	0.039	0.243	0.404	–0.56	0.038	–0.46	0.101	–0.52	0.054
	SO_2_	–0.49	0.075	0.09	0.751	–0.68	0.007	–0.64	0.015	–0.49	0.074
	O_3_	0.68	0.008	0.34	0.237	0.40	0.159	0.41	0.14	0.71	0.005
	CO	–0.49	0.078	–0.02	0.936	–0.55	0.043	–0.49	0.076	–0.55	0.04
Total	PM10	–0.72	0.003	–0.22	0.45	–0.69	0.006	–0.62	0.019	–0.61	0.021
	PM2.5	0.55	0.162	0.26	0.54	0.69	0.059	0.61	0.111	0.73	0.04
	NO_2_	–0.77	0.001	–0.25	0.398	–0.72	0.003	–0.62	0.019	–0.64	0.014
	SO_2_	–0.78	0.001	–0.31	0.283	–0.75	0.002	–0.72	0.004	–0.63	0.015
	O_3_	0.73	0.003	0.63	0.016	0.52	0.059	0.46	0.099	0.37	0.194
	CO	–0.76	0.001	–0.25	0.382	–0.76	0.002	–0.63	0.015	–0.56	0.036

Abbreviation: *n*, number of subjects; Corr, correlation

### Associations among body mass index, green space, ambient temperature, and air pollution

The heat maps presented in Fig. 5-1, 5-2 provide a comprehensive review of the correlations between variables at a glance. For the normal weight group, all cities except Gwangju exhibited a negative correlation between BMI and green space and between BMI and air pollution. In contrast, a positive correlation was observed between BMI and ambient temperature and green space and air pollution. In Gwangju, there was a positive correlation between BMI and green space, BMI and ambient temperature, and green space and ambient temperature, while a negative correlation was observed between BMI and air pollution and green space and air pollution.

**Fig. 5-1:**
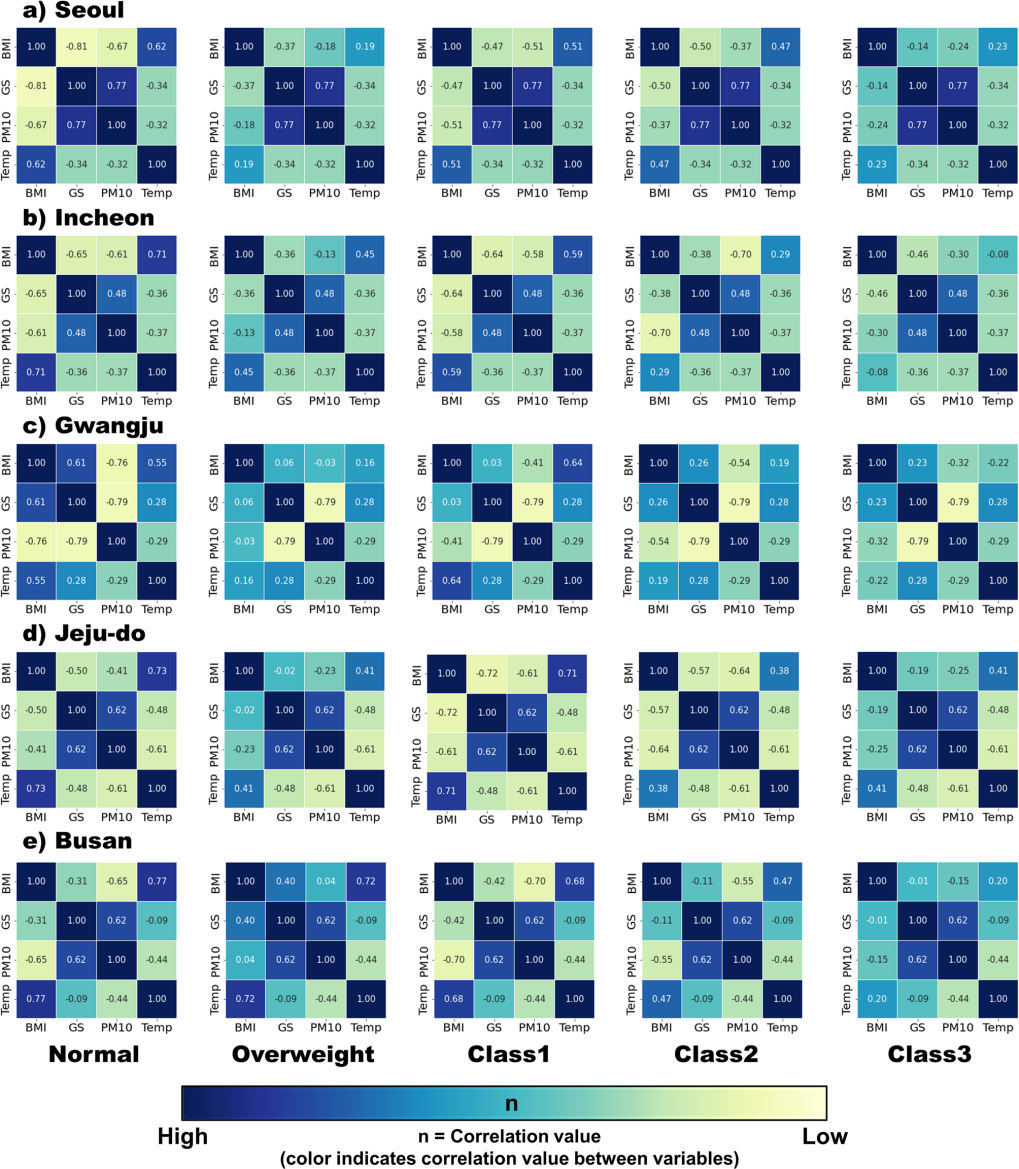
Heat maps of body mass index, green space, ambient temperature, and air pollution. The colors in each numbered box represent the correlation value between the respective variables in columns and rows. Abbreviation: BMI, body mass index; GS, green space; PM, particulate matter; Temp, temperature

**Fig. 5-2:**
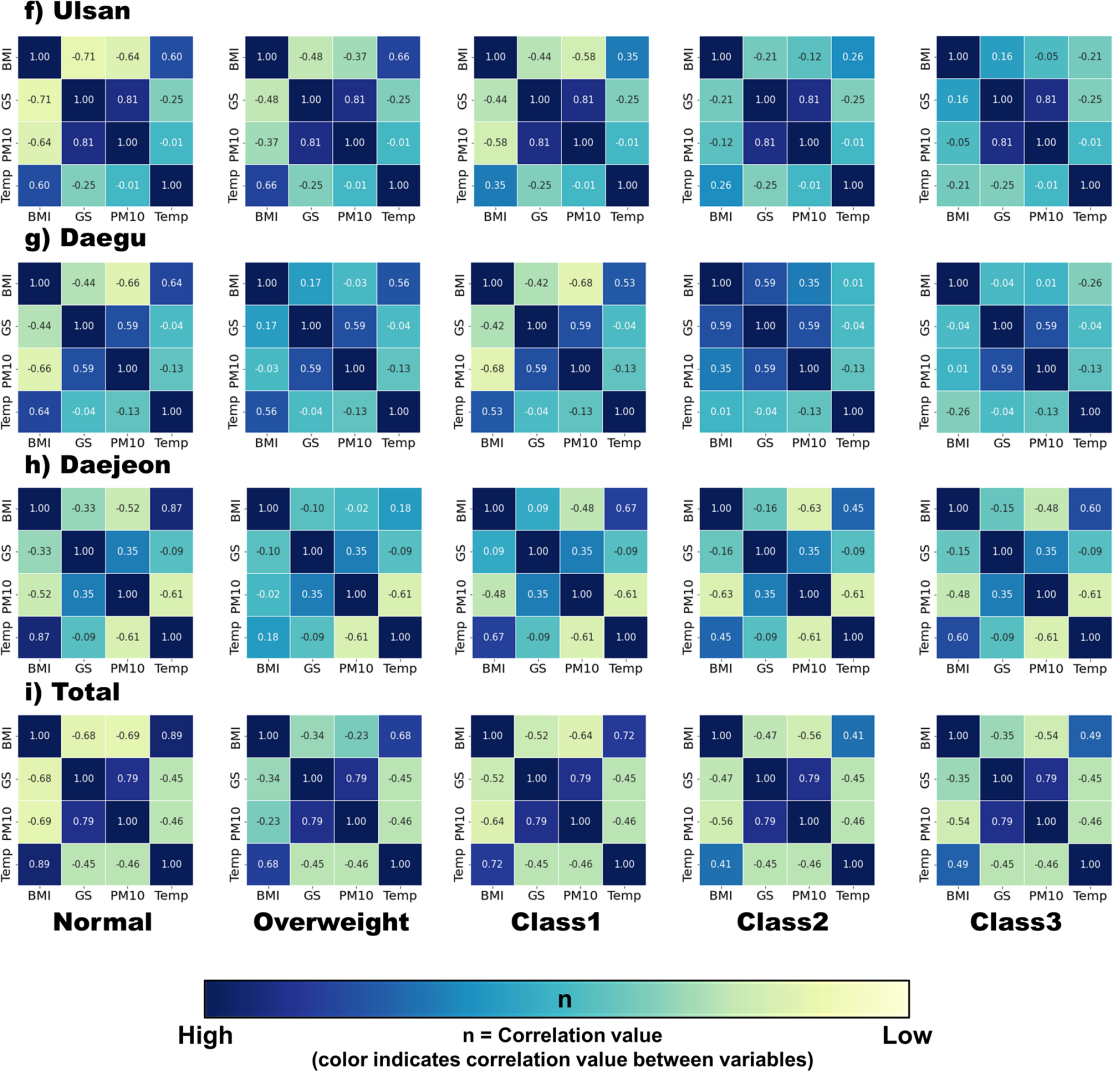
Heat maps of body mass index, green space, ambient temperature, and air pollution. The colors in each numbered box represent the correlation value between the respective variables in columns and rows. Abbreviation: BMI, body mass index; GS, green space; PM, particulate matter; Temp, temperature

In the overweight group, most cities exhibited patterns similar to those in the normal weight group, although some cities, such as Jeju-do, Busan, Daegu, and Daejeon, showed slightly weaker but positive correlations between BMI and green space. The remaining cities showed negative correlations between BMI and green space, similar to the normal weight group. Gwangju showed unique patterns in green space and air pollution, which is identical to the normal weight group in Gwangju but was not observed in other cities.

For all the obesity class groups, the heat map patterns were largely similar across all cities. Most cities in all the obesity class groups exhibited a recognizable positive correlation between green space and air pollution, with the exception of Gwangju, which showed an inverse relationship. As for BMI and ambient temperature, most cities showed a decreasing correlation between BMI and ambient temperature. Some cities, such as Incheon, Gwangju, Ulsan, and Daegu, even showed negative correlations as the BMI increased. A positive correlation between BMI and green space and green space and temperature, and negative correlation between green space and air pollution was only found in Gwangju.

## Discussion

In this study, we aimed to examine the trends and correlations between BMI, green space, ambient temperature, and air pollution from 2008 to 2021 in eight South Korean cities. First, the demographic analysis revealed that while mean age did not vary significantly by BMI, the group with the highest obesity rate had the lowest mean age. Though females constituted a higher percentage of the study population overall, the gender difference was minor in the overweight and obese groups. In fact, the obesity class 1 group included more males than females, reflecting the recent upward trend of male obesity rates in South Korea [14].

Second, the relationship between green space and BMI was inconclusive. Green space decreased over the study period in most cities except Gwangju, whereas BMI levels increased, particularly in the normal weight and obesity class 1–2 groups. In line with our hypothesis, we observed a negative correlation between green space and BMI in the normal weight group across the cities; however, this relationship was not found in the overweight and obesity class 3 groups. By city, only Seoul, Incheon, and Ulsan showed a negative correlation between green space and BMI in the normal weight group, while Jeju-do showed the same correlation in the obesity class 1–2 groups. Interestingly, contrary to prior studies and our hypothesis, Gwangju and Daegu exhibited positive correlations between green space and BMI in the normal weight group and in the obesity class 2 group. This suggests that other factors may influence the green space-BMI relationship.

The lack of a clear correlation between green space and BMI aligns with past studies, which have reported inconsistent findings due to varying population demographics, green space definitions, and national contexts [15–17]. This is believed to be largely due to the lack of precise biopsychosocial pathways that underlie the association between green space and obesity. However, various hypotheses suggest that green space promotes physical activity, social interaction, and psychological well-being, which may reduce obesity rates [15]. Therefore, policymakers should consider features of green space, residential proximity, and amenity of the space when planning urban greenery [16]– [17].

Ambient temperature is another variable that showed an increasing trend in most cities across all BMI groups. Our results revealed positive correlations between ambient temperature and BMI in the normal weight, overweight, and obesity class 1 groups. This supports our hypothesis that higher temperatures may affect BMI through adaptive thermogenesis via brown adipose tissue (BAT) activity [18]– [19]. Adaptive thermogenesis is defined as the complex response of homoeothermic organisms to increase the rate of energy expenditure above normal baseline levels during exposure to cold in order to maintain core temperature [18]. Studies have shown that increased time spent in a thermal neutral zone– the range of ambient temperatures at which the metabolic rate is minimal– can lead to loss of BAT activity, reduced thermogenic capacity, and thus decreased energy expenditure, potentially leading to obesity [19].

Exposure to ambient air pollution has been suggested as a potential contributor to obesity by causing metabolic disorders through inflammatory pathways to initiate metabolic processes [20]. Additionally, epidemiological studies have suggested that air pollution may discourage physical activity and lead to sedentary behaviors [21]– [22]. Nevertheless, our findings indicate a weak link between air pollution and BMI. The majority of cities showed a negative correlation between BMI and levels of PM10, NO_2_, SO_2_, and CO in the normal weight group and obesity class 1–3 groups. These results imply that ambient air pollution may not be a significant contributor to obesity in South Korea. Future research could consider different air pollution measures or timeframes to identify more relevant relationships between air pollution and obesity.

Third, our study investigated how green space, ambient temperature, and air pollution interact. While green space generally declined, with the exception of Gwangju, ambient temperature increased across cities, potentially due to reduced shading and cooling from greenery. Greenery can influence ambient temperature through shading, transpiration, and modified convection. Shading produces cooling by intercepting radiation that would otherwise be absorbed by the surface; transpiration produces cooling by the evaporation of water through plant stomata; and convective heat transfer can modify air movement and increase surface roughness [23]. The gradual decrease in green space may be a reflection of South Korea’s continuous urbanization, which is anticipated to further decrease green spaces in major cities. Our findings highlight a negative correlation between green spaces and ambient temperature, suggesting that diminishing greenery leads to rising temperatures. Furthermore, given the established positive correlation between ambient temperature and BMI within normal weight, overweight, and obesity class 1 group, rising temperatures due to reduced green spaces could indirectly contribute to health challenges in populations considered relatively healthy. This underscores the importance of integrating urban greening initiatives to mitigate temperature-related health risks related to obesity.

Yet, the relationship between green space and air pollution was ambiguous; contrary to studies suggesting that green space reduces air pollution [24]– [25], our data showed positive correlations between green space and air pollution in most cities and BMI groups, with the exception of Gwangju. We speculate that the complex dynamics between air pollution and greenery like green space density, air movement, and transboundary pollution may affect this relationship [26–28]. To better understand this complexity, it is essential to consider the characteristics and sources of the air pollutants included in our anlysis. A wide range of air pollutants, including SO_2_, NO_2_, O_3_, and CO, and commonly measured particulate matters of PM10 and PM2.5, were included in our study. These air pollutants originate from different sources, which vary depending on their type and the urban context. For example, PM10s are usually generated from construction sites, unpaved roads, and industries, while PM2.5s are from sources such as vehicle exhaust, burning of fossil fuels, and cooking with solid fuels [26]. Additionally, South Korea is greatly affected by transboundary air pollution, most notably the yellow dust from northern China and the Mongolian deserts, which contain more PM10 than PM2.5 [27]. These unique characteristics of the air pollutants may have resulted in contrasting trends, with PM10 showing a declining trend and PM2.5 showing an increase throughout the study period.

Furthermore, the rate at which these particulate matters are filtered by the green space depends on the type of urban greenery, surface area, density, humidity, and dynamics of local air movement [28]. Green space can either decrease the local air quality by inhibiting the dispersion of particles from air pollution sources or improve air quality by preventing the arrival of particles emitted elsewhere. Slight changes to the exposure assessment data, exposure metrics, study period, study locations, and other variables regarding green space and air pollution may result in dissimilar results of the relationship between the two.

As shown in the heat maps, the relationship between ambient temperature and air pollution in our study revealed a negative correlation in all cities across all BMI groups. This finding contrasts with the consensus that ambient temperature and air pollution may have synergetic effects between the two; heat triggers primary pollutant emissions, transforms and worsens air pollutants through chemical reactions, and causes high atmospheric pressure that keeps air pollution at the ground level leading to a stagnant environment [29]. As such, global warming and air pollution combine into a major public health concern, which includes cardiovascular, pulmonary, and metabolic diseases.

This negative correlation observed in our study likely reflects successful air quality initiatives in cities, which could have resulted in lower levels of pollution despite higher temperatures. A closer look at the specific trends of these pollutants such as increases in O_3_ and PM2.5 suggest that further analysis is needed to understand the link between rising temperatures and pollution. If O_3_ and PM2.5 are prioritized for further analysis, the findings in our study align with prior studies that show the strongest evidence of synergistic effects between ambient temperature and air pollution [30]. This relationship is particularly relevant to obesity, as both O_3_ and PM2.5 have been identified as contributors to metabolic dysfunction through multiple pathways. Exposures to these pollutants can initiate oxidative stress and inflammation, disrupt metabolic processes, and promote fat accumulation, leading to increased risks of metabolic disorders and weight gain. Additionally, air pollution can trigger epigenetic changes, including modifications to DNA methylation, histones, and noncoding RNA expression, which affect key biological processes such as inflammation, oxidative stress regulation, and cellular signaling [31]. These pathways suggest that individuals in areas with rising temperatures and persistent levels of PM2.5 or O_3_ may face heightened risks of obesity-related health issues.

Finally, our study has several limitations. First, the inclusion of only eight cities in our study may not represent all of South Korea. Adding more cities from the eastern coastal area could provide a more complete picture. Second, more refined methods to measure ambient temperature and air pollution across urban and rural areas would help capture a broader range of exposures. The monitoring weather stations, and air quality monitoring system (Air Korea) stations are generally sparse and concentrated around urban areas, making it difficult to examine rural populations. Lastly, our study only examined parks as green spaces to facilitate statistical analysis and emphasize their role in promoting physical activity. Future studies could broaden the scope of green spaces by including different types of green spaces, allowing for a more comprehensive understanding of the relationship between green space and health outcomes.

## Conclusion

In conclusion, this study investigated the relationships between BMI, green space, ambient temperature, and air pollution in eight South Korean cities from 2008 to 2021.

### Main findings

The demographic analysis highlighted the need for gender-specific interventions for obesity. The investigation into green space and BMI revealed inconclusive results, aligning with previous research challenges in establishing a definitive link. On the other hand, the escalating ambient temperature displayed positive correlations with BMI, supporting the role of adaptive thermogenesis in obesity dynamics. Contrary to expectations, the weak link observed between air pollution and BMI in South Korea also suggests that there may be intricate factors that influences the observed association between air pollution and BMI.

The interactions between green space, ambient temperature, and air pollution yielded heterogeneous results, emphasizing the complexity of these relationships. While the declining green space likely contributed to increased ambient temperature, the correlation between green space and air pollution remained ambiguous, warranting further exploration.

This study makes several meaningful contributions to the existing body of literature on environmental determinants of obesity. First, the use of a 14-year longitudinal dataset enables us to examine long-term trends and the evolving relationship between environmental change and obesity with greater precision than studies based on shorter time spans. Second, while recent works such as Tu et al. (2024) have explored similar themes through literature reviews, our study is distinguished by its empirical, data-driven approach that provides robust quantitative evidence [32]. Third, by focusing on urban areas in South Korea, we shed light on the public health impacts of urbanization—particularly air pollution, thermal environmental stress, and the reduction of green spaces—on obesity, thereby offering insights relevant to policy and urban planning. Fourth, our city-level analysis enables meaningful cross-city comparisons, offering a novel lens through which to understand how local environmental and policy contexts shape public health outcomes.

Moreover, the inclusion of health information from a large sample of 674,962 individuals enhances the generalizability and reliability of our findings. Taken together, these novel aspects position our study as a significant advancement in understanding the complex relationship between environmental factors and obesity in urbunized contexts.

### Future suggestions

The study’s limitations underscore the need for more comprehensive future research, encompassing a broader range of cities, refined measurement methods for ambient temperature and air pollution, and a more inclusive definition of green space. Furthermore, expanding the scope of green spaces beyond parks may be crucial for a holistic understanding of their impact on physical activity and overall well-being. Addressing these limitations will contribute to a more refined comprehension of the dynamics shaping the relationship between urban environments and obesity.

## Data Availability

The data that support the findings of this study are available from Korea Disease and Prevention Agency, Korean Statistical Information Service, Korea Meteorological Administration, and Korea Environment Corporation. But restrictions apply to the availability of these data, which were used under license for the current study, and so are not publicly available. Data are however available from the authors upon reasonable request and with permission of Korea Disease and Prevention Agency, Korean Statistical Information Service, Korea Meteorological Administration, and Korea Environment Corporation (Corresponding authors: cjun@korea.ac.kr).

## Abbreviations

ASOS: Automated Synoptic Observation System
BMI: Body Mass Index
CO: Carbon Monoxide
KDCA: Korea Disease Control and Prevention Agency
KCHS: Korea Community Health Survey
NO_2_: Nitrogen Dioxide
O_3_: Ozone
PM: Particulate Matter
SO_2_: Sulfur Dioxide
